# Liraglutide reduces lipid accumulation in steatotic L-02 cells by enhancing autophagy

**DOI:** 10.3892/mmr.2014.2569

**Published:** 2014-09-15

**Authors:** SHI-WEI ZHOU, MAN ZHANG, MIN ZHU

**Affiliations:** Department of Geriatrics, Huashan Hospital, Fudan University, Shanghai 200040, P.R. China

**Keywords:** non-alcoholic fatty liver disease, glucagon-like peptide-1, autophagy, hepatic steatosis, liraglutide

## Abstract

Simple hepatic steatosis is the early stage of non-alcoholic fatty liver disease and is recognized as a benign process. Previous studies show that glucagon-like peptide-1 has great potential in improving hepatic steatosis. Recent data have revealed that inhibiting autophagy exacerbates lipid accumulation in hepatocytes. Therefore, the present study aimed to determine the effects of liraglutide (LG) on simple hepatic steatosis and the possible role of autophagy. Firstly, steatotic L-02 cells were induced by incubating L-02 cells with 1 mmol/l free fatty acid (FFA) mixture (oleic acid and palmitic acid at a molar ratio of 2:1) for 24 h. Intracellular lipid accumulation, cell viability, oxidative stress and apoptosis were evaluated. Secondly, steatotic L-02 cells were treated with 10 or 100 nmol/l LG, 100 nmol/l LG plus 3-methyladenine (3-MA), or rapamycin for 24 h, and then lipid accumulation was measured. Next, the degree of lipid accumulation and the intensity of autophagy were assessed. Oil red O staining and triglyceride quantification demonstrated notable steatosis in L-02 cells following exposure to 1 mmol/l FFA mixture for 24 h. There was no significant cytotoxicity, oxidative stress or apoptosis in steatotic L-02 cells. Treatment with 100 nmol/l LG reduced lipid accumulation in steatotic L-02 cells and increased the mRNA levels of microtubule-associated protein 1 light chain 3B. Additionally, it enhanced the autophagic flux in steatotic L-02 cells, as measured by western blot analysis and shown by electron microscopy. Additionally, 3-MA weakened the ability of LG to improve hepatic steatosis and enhance autophagy. Our data indicate that LG reduces the lipid accumulation in steatotic L-02 cells, and the activation of autophagy plays a significant role in this process.

## Introduction

Non-alcoholic fatty liver disease (NAFLD) is currently the most common form of chronic liver disease affecting both adults and children. It is strongly associated with obesity, diabetes and hyperlipidemia. Thus, NAFLD is often considered as the hepatic manifestation of metabolic syndrome. NAFLD comprises a pathological spectrum ranging from simple hepatic steatosis and non-alcoholic steatohepatitis (NASH) to liver fibrosis, cirrhosis and hepatocellular carcinoma (1). Although previously thought to be benign, simple hepatic steatosis can evolve into more severe liver diseases (2). In addition, individuals with simple hepatic steatosis are at an increased risk of cardiovascular disease, diabetes and obesity-related and overall mortality (3). Aside from lifestyle modification, there is currently no effective therapy for simple hepatic steatosis (2).

Glucagon-like peptide-1 (GLP-1) is an incretin secreted by L-cells in the small intestine in response to food intake, and is rapidly degraded by dipeptidyl peptidase 4 present on the endothelial cells lining capillaries of the lamina propria. The main roles of GLP-1 in the pancreas are the stimulation of glucose-dependent insulin secretion, inhibition of postprandial glucagon release and induction of β-cell proliferation (4). Apart from the pancreatic islets, GLP-1 receptors (GLP-1Rs) are also present in several other tissues including the nervous system, gastrointestinal tract, lung and heart. As a result, GLP-1 exerts further beneficial functions including delaying stomach emptying, increasing satiety, reducing food intake and triggering weight loss (5). Recently, two studies have suggested that GLP-1Rs exist in the liver (6,7). Accumulating evidence reveals that GLP-1-related drugs reduce hepatic steatosis both *in vivo* and *in vitro* (6–10). A few studies in humans have also proven that utilization of GLP-1R agonists can improve hepatic steatosis, particularly in type 2 diabetes patients with NAFLD (as reviewed in 11). However, until now, there has been little research into the effect of GLP-1 on simple hepatic steatosis.

Autophagy is a highly evolutionarily conserved process involved in the turnover of long-lived proteins, cytosolic components or damaged organelles. Three types of autophagy are known: Macroautophagy, chaperone-mediated autophagy and microautophagy. Furthermore, macroautophagy (hereafter referred to as autophagy) is the main regulated catabolic mechanism used by eukaryotic cells to degrade long-lived proteins and organelles. It is well known that autophagy is widely involved in the pathogenesis of numerous diseases and processes including infections, cancer, neurodegeneration, aging and heart disease (12). Previous studies have discovered new functions for autophagy in the regulation of lipid metabolism. The inhibition of autophagy by genetic knockdown of the autophagy gene *ATG*5, or treatment with 3-methyladenine (3-MA) in cultured hepatocytes that were challenged with a lipid load from fatty acid supplementation, can significantly increase intracellular triglyceride (TG) content (13). Another study reported that increased steatosis in models of alcohol-induced hepatic injury can be induced by inhibition of autophagy (14). These studies indicate that modulating autophagy may be a new therapeutic strategy for NAFLD.

Until now, relatively few studies have investigated whether GLP-1 reduces lipid accumulation through modulating autophagy. With this background, we hypothesize that GLP-1 is able to improve simple hepatic steatosis by enhancing autophagy. Therefore, in the present study, we selected the L-02 cell line, which is an immortalized normal human hepatocyte-derived cell line, to construct a cell model of simple hepatic steatosis. Subsequently, we used liraglutide (LG), a long-acting GLP-1 analog, to treat the cell model and measure the change of intracellular lipid accumulation. Furthermore, we detected the intracellular level of autophagy by utilizing autophagic tool drugs, which modulate the activity of autophagy, including autophagy inhibitors and activators, and LG.

## Materials and methods

### Cell culture

L-02 cells (Institute of Biochemistry and Cell Biology, Shanghai Institute for Biological Sciences, Shanghai, China) were incubated in Dulbecco’s modified Eagle’s medium (DMEM; Invitrogoen Life Technologies, Carslbad, CA, USA) supplemented with 10% fetal bovine serum and 1% penicillin/streptomycin (Invitrogoen Life Technologies) at 37°C in an atmosphere containing 5% CO_2_. Cells were used for experiments at 75% confluency.

### Steatotic hepatocyte model construction

Stock solution of oleic acid (OA; Sigma-Aldrich, St. Louis, MO, USA) and palmitic acid (PA; Sigma-Aldrich) were prepared as described previously (15). In brief, a 15-mmol/l solution of OA or PA in 0.01 mmol/l NaOH was incubated at 70°C for 30 min, and then fatty acid soaps were mixed with 5% bovine serum albumin (BSA; Millipore, Billerica, MA, USA) in phosphate-buffered saline (PBS) (the molar ratio of fatty acid to BSA was 7:1). To induce steatosis, L-02 cells were exposed to 1 mmol/l free fatty acid (FFA) mixture (OA:PA ratio, 2:1). After incubation for another 24 h, cells were used for later analysis or experiments.

### Experimental grouping

To investigate the role of autophagy in the treatment of simple hepatic steatosis by LG (donated by Novo Nordisk, Bagsvaerd, Denmark), L-02 cells were firstly exposed to 1 mmol/l FFA mixture for 24 h and then divided into six groups to be exposed to the following stimulation: i) saline (model group), ii) 10 nmol/l LG (LG10 group), iii) 100 nmol/l LG (LG100 group), iv) 100 nmol/l LG + 3-MA (autophagic inhibitor, 5 mmol/l; Sigma-Aldrich) (negative control, LG100M group), v) rapamycin (RAPA, autophagic enhancer, 1 μmol/l; Sigma-Aldrich) (positive control, RAPA group), vi) incubated in DMEM for 24 h and then given saline (normal group). After another 24 h, cells were collected for further analyses including oil red O staining, intracellular TG quantification, quantitative polymerase chain reaction (qPCR), western blot analysis and electronic microscopy. Furthermore, to assess the autophagic flux, L-02 cells were exposed to 100 nmol/l bafilomycin (Sigma-Aldrich) following the aforementioned treatments for 24 h. After 24 h, cell lysates were quantified by western blot analysis. The differences in the amount of microtubule-associated protein 1 light chain 3B (LC3B)-II between groups in the presence and absence of bafilomycin represent the amount of LC3B-II that is delivered to lysosomes for degradation (16).

### Oil red O staining

To observe the lipid droplets in the L-02 cells that were cultivated in the six-well plates, each group was processed with the relevant treatment then rinsed three times with PBS, fixed in 4% paraformaldehyde for 30 min, stained for 15 min at room temperature in freshly diluted oil red O, decolorized for 15 sec in 70% ethanol solution, redyed for 30 sec in hematoxylin staining solution and rinsed with PBS twice. Finally, intracellular lipid droplets were observed and captured with an inverted microscope (Mode DIMRE2; Leica, Wetzlar, Germany) connected to a digital camera (Mode DP70; Olympus, Tokyo, Japan_.

### Intracellular TG quantification

Intracellular TG quantification assay was performed according to the manufacturer’s instructions (K622-100; Biovision, Milpitas, CA, USA).

### Cell viability assay

Cell viability was assessed by the Cell Counting Kit-8 (CCK-8) assay (Dojindo Molecular Technologies, Inc., Kumamoto, Japan). L-02 cells were treated with 1 mmol/l FFA in 96-well plates. Following incubation for 24 h, 10 μl CCK-8 solution was added to each well and the plates were further incubated at 37°C for 1–2 h. Subsequently, the optical densities of the plates were read on a microplate reader (model 3550; Bio-Rad Laboratories, Inc., Hercules, CA, USA) using a test wavelength of 450 nm. The absorbance was directly proportionate to the number of living cells.

### Evaluation of intracellular oxidative stress

Malondialdehyde (MDA) is a marker of oxidative stress. MDA content was evaluated using an intracellular MDA quantification kit (A003-4; NanJing JianCheng Bioengineering Institute, Nanjing, China).

### Apoptosis analysis

Early- and late-phase apoptotic cells were monitored with an Annexin V-fluorescein isothiocyanate (FITC) apoptosis detection kit (KGA108; Nanjing KeyGen Biotech Co., Nanjing, China). Following treatment with 1 mmol/l FFA, L-02 cells were harvested, washed twice with cold PBS, resuspended in binding buffer and incubated with Annexin V-FITC and propidium iodide (PI) staining solution following the manufacturer’s instructions. Samples of 10,000 stained cells were analyzed using a flow cytometer (BD Biosciences, San Jose, CA, USA).

### qPCR

Total RNA was extracted from L-02 cells and converted to cDNA using the PrimeScript RT reagent kit (Takara Bio, Inc., Shiga, Japan). LC3B mRNA levels were measured by qPCR with the ABI Prism 7900 sequence detection system (Life Technologies, Carlsbad, CA, USA), with β-actin as an internal reference gene. The reaction mixture contained 0.1 mol/l of each primer, 2X SYBR-Green PCR Master mix (Takara Bio., Inc.) and 1 μl cDNA (1:10 dilution). Each reaction was performed in triplicate. The primers were designed as follows: Sense, 5′-CAACTGGGACGACATGGAGAAAAT-3′ and anti-sense, 5′-CCAGAGGCGTACAGGGATAGCAC-3′ for β-actin; sense, 5′-GAGCAGCATCCAACCAAAAT-3′ and anti-sense, 5′-CTGTGTCCGTTCACCAACAG-3′ for LC3B. The relative mRNA expression levels were quantified using the 2^−ΔΔCt^ method with β-actin as an endogenous control (16).

### Western blot analysis

L-02 cells in each 25-cm^2^ culture flask were collected and proteins were extracted with 1 ml mixture of RIPA lysis buffer (P0013B; Beyotime Institute of Biotechnology, Haimen, China) and phenylmethylsulfonyl fluoride (final concentration, 1 mmol/l). Protein concentration was determined using a bicinchoninic acid protein assay kit (P0012; Beyotime Institute of Biotechnology). Thirty micrograms of protein samples were separated by SDS-PAGE and then electro-transferred onto the polyvinylidene difluoride membranes (Millipore Corporation, Billerica, MA, USA). Membranes were blocked for 1 h with 5% skimmed milk in TBST buffer [50 mmol/l Tris (pH 7.6), 150 mmol/l NaCl and 0.1% Tween-20] and incubated with rabbit monoclonal antibodies against human LC3B (Cell Signaling Technology, Inc., Beverly, MA, USA) overnight at 4°C. β-actin was used as an internal control and was detected using a mouse monoclonal antibody against β-actin (Sigma, St. Louis, MO, USA). Following incubation with secondary antibodies, enhanced chemiluminescence detection reagents (Beyotime Institute of Biotechnology) were used to detect the signals. The intensity of the signals was quantified with Image Lab 3.0 software (Bio-Rad Laboratories, Inc.).

### Electron microscopy

Following administration of the relevant treatment, L-02 cells were fixed with 2% glutaraldehyde in 0.1 mol/l phosphate buffer (pH 7.4) followed by 1% OsO4. After dehydration, thin sections were cut and stained with uranylacetate and lead citrate. Digital images were obtained using a JEM1200EX transmission electron microscope.

### Statistical analysis

All data points are expressed as the mean ± standard error. For comparison of two groups, the unpaired Student’s t-test was used. In instances of multiple means comparisons, one-way analysis of variance followed by Bonferroni post hoc tests were used. P<0.05 was considered to indicate a statistically significant difference. Graphs and statistical analyses were generated using Graphpad Prism 5 for Windows (Graphpad Software, Inc., La Jolla, CA, USA).

## Results

### FFA treatment induced lipid accumulation in L-02 cells

**Figure 1:**
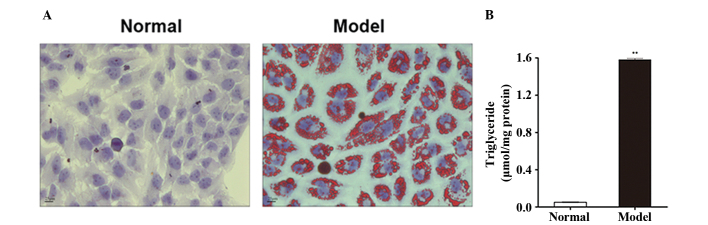
Lipid accumulation induced by 1 mmol/l free fatty acid (FFA) mixture in L-02 cells. (A) L-02 cells were exposed to 1 mmol/l FFA mixture (oleate and palmitate at a ratio of 2:1) for 24 h. Lipid accumulation was analyzed by oil red O staining. Original magniﬁcation, ×40. (B) Intracellular triglyceride levels were quantified. Normal, normal group; model, model group. Results are expressed as the mean ± SE of three independent experiments. ^**^P<0.01, versus the normal group.

L-02 cells were incubated in DMEM containing 1 mmol/l FFA mixture (OA:PA ratio, 2:1) for 24 h and then stained with oil red O. We observed a large quantity of red lipid droplets in L-02 cells in the model group (Fig. 1A). However, no lipid droplets were detected in L-02 cells in the normal group (Fig. 1A). As shown in Fig. 1B, the intracellular TG level was significantly higher in the model group than in the normal group (P=3.28×10^−8^).

### FFA treatment did not induce significant cytotoxicity, oxidative stress or apoptosis

**Figure 2:**
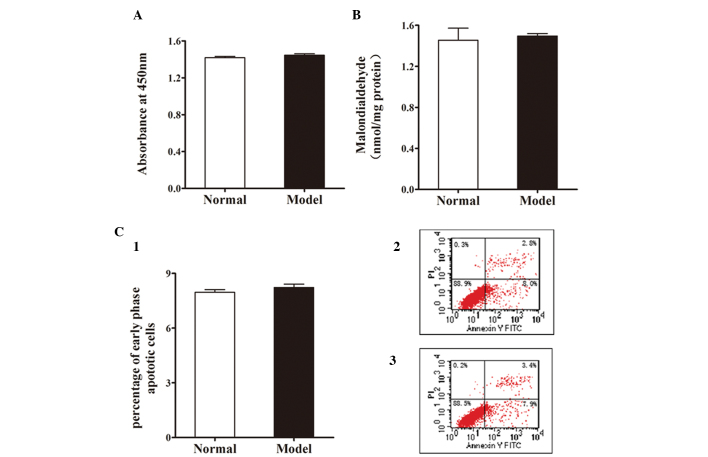
Effects of cytotoxicity, oxidative stress and apoptosis on L-02 cells after free fatty acid (FFA) stimulation. (A) L-02 cells were cultured in DMEM containing 1 mmol/l FFA mixture (oleate and palmitate at a ratio of 2:1) for 24 h. Cell viability was determined by the Cell Counting Kit-8 assay. Absorbance at 450 nm was positively correlated with cell viability. (B) Malondialdehyde, a widely-used marker of intracellular oxidative stress, was quantified in the cells of the normal group and model group. (C) L-02 cells were exposed to 1 mmol/l FFA mixture for 24 h and then stained with Annexin V-fluorescein isothiocyanate and propidium iodide. Normal, early apoptotic, late apoptotic and necrotic cells are presented in the lower left, lower right, upper right and upper left quadrants, respectively. Results are representative of three independent experiments. (1) Quantification of early phase apoptotic cells in response to FFA treatment; (2) normal group; (3) model group.

L-02 cells were treated with 1 mmol/l FFA mixture for 24 h and the cytotoxicity of FFA to L-02 cells was analyzed by CCK-8 assay. According to the manufacturer’s instructions, absorbance at 450 nm was positively correlated with cell viability. Fig. 2A reveals no significant difference between the normal group and the model group in cell viability.

There was no significant increase in MDA content, a widely used marker of intracellular oxidative stress, following FFA treatment (Fig. 2B).

To evaluate the apoptotic effect of FFA treatment on L-02 cells, the cells were incubated with 1 mmol/l FFA mixture for 24 h and then stained with Annexin V-FITC/PI. Apoptosis was measured by flow cytometry. As shown in Fig. 2C, FFA treatment had no apparent effect on early phase apoptosis in L-02 cells.

These data demonstrate that it is possible to construct a steatotic hepatocyte model by incubating L-02 cells with 1 mmol/l FFA mixture (OA:PA=2:1) for 24 h. Notable lipid accumulation without apparent cytotoxicity, oxidative stress or apoptosis was detected in this cell model, which was similar to that observed in simple hepatic steatosis in humans by Farrell and Larter (17).

### LG reduced lipid accumulation in steatotic L-02 cells

**Figure 3:**
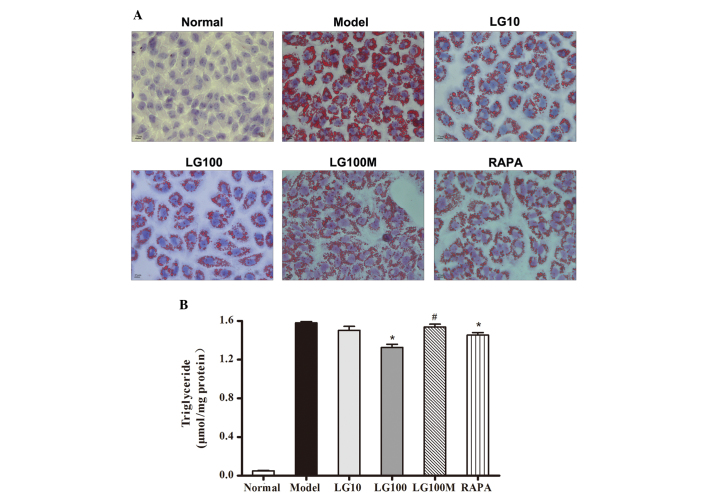
Liraglutide reduced lipid accumulation in free fatty acid (FFA)-loaded L-02 cells. (A) L-02 cells were incubated with 1 mmol/l FFA mixture for 24 h and then given various treatments. After another 24 h, L-02 cells were stained with oil red O. Original magnification, ×40. (B) Intracellular triglyceride was quantified. Normal, normal group; model, model group; LG10, 10 nmol/l liraglutide group; LG100, 100 nmol/l liraglutide group; LG100M, 100 nmol/l liraglutide + 5 mmol/l 3-methyladenine (autophagic inhibitor) group; RAPA, 1 μmol/l rapamycin (autophagic enhancer) group. Results are expressed as the mean ± SE from three independent experiments. ^*^P<0.05, versus the model group; and ^#^P<0.05, versus the LG100 group.

To determine the effect of LG on lipid accumulation in FFA-treated L-02 cells and the role of autophagy in this process, steatotic L-02 cells were given various treatments and the lipid accumulation was quantified, the results of which were further confirmed using oil red O staining (Fig. 3A). As shown in Fig. 3B, 10 and 100 nmol/l LG both reduced lipid accumulation in steatotic L-02 cells, and the difference between the model group and the LG100 group was significant (P=1.67×10^−4^). Compared with the use of 100 nmol/l LG alone, use of both LG (100 nmol/l) and 3-MA (autophagic inhibitor) increased the intracellular lipid accumulation (Fig. 3B; P=9.83×10^−4^). Using RAPA (autophagic enhancer) alone also reduced the lipid accumulation in the L-02 cells (P=0.013).

### LG enhanced autophagy in steatotic L-02 cells

**Figure 4:**
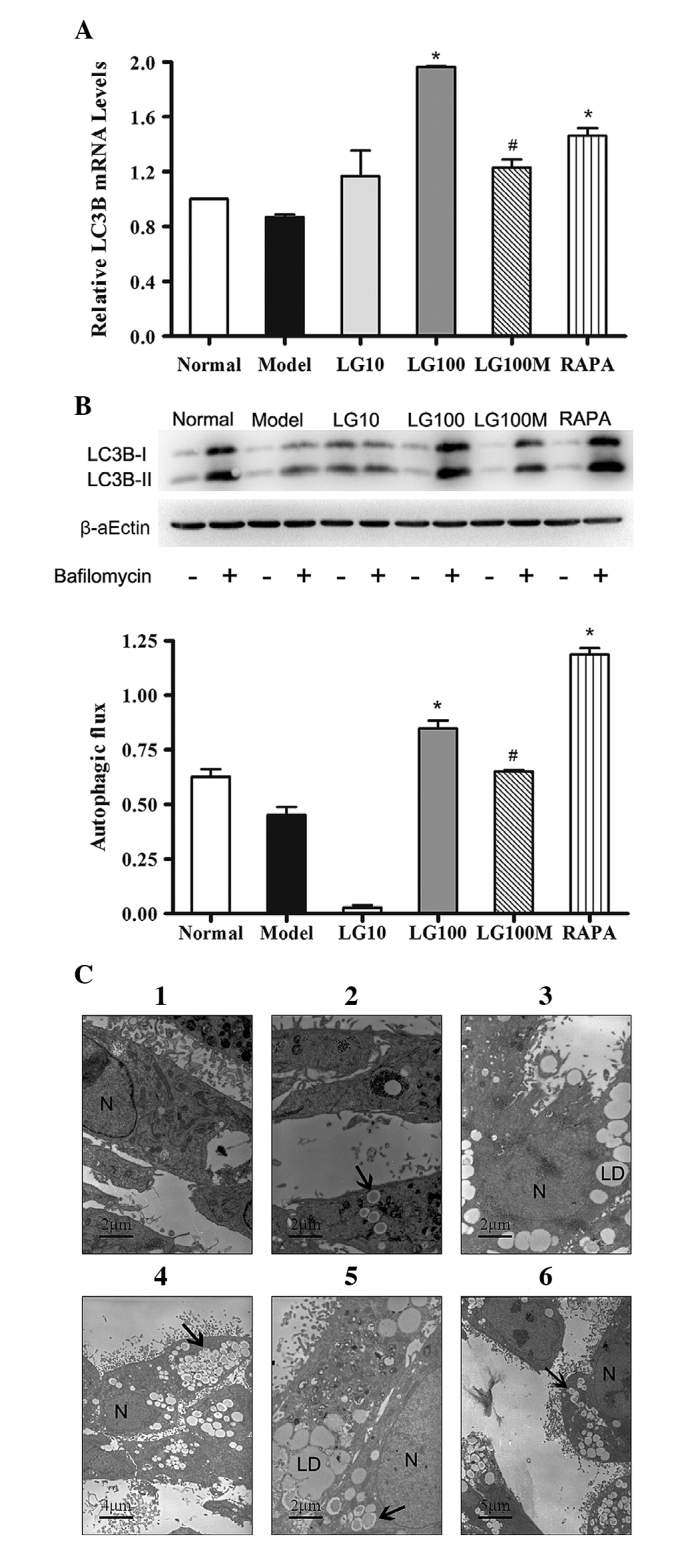
Liraglutide enhanced autophagy in L-02 cells treated with FFA mixture for 24 h. (A) LC3B mRNA expression level in the groups given various treatments. β-actin was used as a loading control. (B) Immunoblot for LC3B in the respective groups in the presence or absence of bafilomycin (100 nmol/l). Autophagic flux is depicted as the values obtained by subtracting the densitometry values of LC3B-II in bafilomycin-free samples from their respective bafilomycin-treated counterparts. Normal, normal group; model, model group; LG10, 10 nmol/l liraglutide group; LG100, 100 nmol/l liraglutide group; LG100M, 100 nmol/l liraglutide + 5 mmol/l 3-methyladenine (autophagic inhibitor) group; RAPA, 1 μmol/l rapamycin (autophagic enhancer) group. Results are expressed as the mean ± SE from three independent experiments. ^*^P<0.05, versus the model control; and ^#^P<0.05, versus the 100 nmol/l liraglutide group. (C) Electron microscopy of L-02 cells in the groups given various treatments. (1) and (2) normal group; (3) model group; (4) 100 nmol/l liraglutide group; (5) 100 nmol/l liraglutide+5 mmol/l 3-MA group; and (6) RAPA, 1 μmol/l rapamycin group. N, nucleus; LD, lipid droplets; black arrows, autophagosomes or autophagolysosomes.

LC3B-II is an important protein marker that is reliably associated with phagophores, sealed autophagosomes and mature autophagosomes/autolysosomes (18). To evaluate the intensity of autophagy in the respective groups, L-02 cells were collected following treatment, and then qPCR, western blot analysis and electron microscopy were conducted. As shown in Fig. 4A, the LC3B mRNA expression levels in the LG100 group were significantly higher than those in the model group (P=1.18×10^−5^). When 100 nmol/l LG and 3-MA were both used, the LC3B mRNA expression levels were notably decreased compared with those in the LG100 group (P=6.73×10^−4^). The LC3B mRNA expression levels in the RAPA group were significantly higher than those in the model group (P=0.0044).

Since LC3B-II is localized both in the lumenal and the cytosolic site of the autophagosome and undergoes rapid degradation within the lysosomes, the increase in LC3B-II may be the consequence of its increased formation and/or its attenuated degradation (18). In the present study, we estimated the autophagic flux by inferring LC3B-II turnover in the presence and absence of bafilomycin (a vacuolar-type H^+^-ATPase inhibitor). The L-02 cells in the respective groups were incubated either alone or with 100 nmol/l bafilomycin. Since bafilomycin inhibits the fusion of autophagosomes to lysosomes, the values obtained by subtracting the densitometry values of LC3B-II in bafilomycin-free samples from their respective bafilomycin-treated counterparts were taken as the autophagic flux (18). Fig. 4B reveals that the autophagic flux in the LG100 group was significantly higher than that in the model group (P=8.11×10^−6^). The same trend was detected in the RAPA group (P=7.87×10^−9^). Compared with the LG100 group, the LG100M group demonstrated a significant decrease in autophagic flux (P=0.0064). These results were in accordance with the change in the LC3B mRNA expression levels.

Electron microscopy is well recognized as the golden standard to observe the autophagosome. Autophagosomes and autolysosomes were difficult to discern as they both present as autophagic vacuoles (AVs). As shown in Fig. 4C (1–2), numerous mitochondria and few AVs were present in normal L-02 cells. Following treatment with 1 mmol/l FFA mixture, many lipid droplets were observed in the cytoplasm around the nucleus, as shown in Fig. 4C (3). A large amount of AVs were observed in steatotic L-02 cells treated with 100 nmol/l LG for 24 h (Fig. 4C-4). In steatotic L-02 cells treated with 100 nmol/l LG and 3-MA, there were numerous lipid droplets and few AVs (Fig. 4C-5). A few AVs and lipid droplets coexisted in cells treated with RAPA (Fig. 4C-6). In summary, treatment with 100 nmol/l LG enhanced the intensity of autophagy in steatotic L-02 cells.

## Discussion

Excess lipid accumulation in hepatocytes is usually recognized as the first step in the progression of NAFLD (17). GLP-1 and related peptides have the potential to reduce lipid content in hepatocytes (11). Autophagy is involved in hepatic lipid metabolism, and intracellular lipid accumulation significantly increases when autophagy is inhibited (13). Our present study demonstrated that LG, a long-acting GLP-1 analog, reduced lipid accumulation in the steatotic L-02 cell model, and could mimic the pathogenic features of NAFLD (known as simple hepatic steatosis) in humans. In addition, our study provides potential mechanistic insights that LG improves hepatic steatosis by enhancing autophagy.

The pathogenesis of NAFLD has not been clarified until now and the ‘two hits theory’ is widely accepted. Excess lipid accumulation in hepatocytes is usually recognized as the first hit (19). PA and OA are the most abundant FFAs in liver TGs in both normal subjects and patients with NAFLD. A previous study showed that human hepatocytes and HepG2 cells can be induced into steatosis by incubating with FFA mixture for 24 h. FFA mixture (1 mmol/l; OA:PA ratio=2:1) is associated with minor toxic and apoptotic effects, thus representing a cellular model of steatosis that mimics benign chronic steatosis (20). However, due to the scarcity of human hepatocytes and the uncertainty in using liver cancer cell lines, such as HepG2 or Huh-7, we chose the normal human hepatocyte-derived cell line L-02 in our study. Steatotic L-02 cells, obtained by incubating L-02 cells with 1 mM FFA for 24 h, had apparent steatosis with minor cytotoxic, oxidative and apoptotic effects. Hence, the steatotic L-02 cell model reproduces the key features of simple hepatic steatosis in human and is suitable for studies on the pathogenesis and treatment of NAFLD.

GLP-1-related drugs were previously used for the treatment of diabetes (21); however, recent studies have revealed the potential function of GLP-1-related drugs in improving NAFLD. Ding *et al* (8) demonstrated that exentin-4, a peptide agonist of GLP-1R, effectively reversed hepatic steatosis in ob/ob mice by improving insulin sensitivity. Gupta *et al* (7) induced steatosis by incubating Huh7 cells with 0.4 mmol/l PA and 0.4 mmol/l OA for 12 h, and then treated the cells with 20 nmol/l exentin-4 for 6 h. A significant reduction in lipid content was observed, and the same tendency in HepG2 cells cultured in methionine-choline-deficient media was revealed. In the present study, we treated steatotic L-02 cells with LG and conducted oil red O staining and intracellular TG quantification. The results revealed a significant reduction in lipid accumulation in steatotic L-02 cells (Fig. 3). Since the steatotic L-02 cell model was able to mimic the features of human simple hepatic steatosis, our results suggest a new role for GLP-1-related drugs in simple hepatic steatosis.

The hallmark of NAFLD is hepatic lipid (mainly TG) accumulation in the absence of significant ethanol consumption, viral infection or other specific etiologies. Hepatic lipid accumulation results from an imbalance between lipid availability (from circulating lipid uptake or de novo lipogenesis) and lipid disposal (via FFA oxidation or very low density lipoprotein secretion) (22). Previous studies have made great efforts to elucidate the mechanism underlying the anti-steatosis effects of GLP-1-related drugs. These studies suggested that GLP-1-related drugs such as exendin-4 were able to reduce lipid accumulation through reducing weight and improving insulin sensitivity (6–8). In addition, there is much evidence to show that GLP-1-related drugs attenuate hepatic steatosis through targeting the hepatic lipid metabolism, including inhibiting lipogenesis (10,23), promoting fatty acid β-oxidation (10,24), increasing fatty acid transport (24) and enhancing lipid droplet fission (25). The phosphorylation of cAMP activated kinase (AMPK) was recognized as being crucial in the above pathways (6,10,23).

Previous studies have implicated a role of autophagy in hepatic lipid metabolism. Škop *et al* (26) suggested that the inhibition of the autophagic flux or lysosomal activity decreased the secretion of very low density lipoprotein and formation of FFA oxidative products, while the stimulation of autophagy by RAPA increased some of these indicators. The study by Singh *et al* (13) demonstrated that the inhibition of autophagy (by pharmacological or genetic approaches) in cultured hepatocytes and mouse liver increased TG storage in lipid droplets. The ablation of liver-specific autophagy led to excessive hepatic lipid accumulation and the development of fatty liver. These findings suggested that the upregulation of autophagy in hepatocytes could increase the breakdown of lipid stores and may serve as a novel approach in treating NAFLD.

The present study demonstrated that LG reduces lipid accumulation in steatotic L-02 cells. The role of autophagy in the anti-steatosis effect of LG was investigated, and the results revealed that LG increased the mRNA and protein expression level of LC3B-II, which serves as a widely used marker for autophagosomes (18). Since autophagy is a dynamic process and LC3B-II is degraded in this process, we further analyzed the autophagic flux. Compared with that in the model group, the intensity of the autophagic flux in the LG (100 nmol/l) group was significantly enhanced. To evaluate autophagy more precisely, electron microscopy was employed to observe autophagosomes and autolysosomes (both taken as AVs). This revealed that the number of AVs was notably increased following treatment with 100 nmol/l LG. 3-MA inhibits autophagosome formation *in vitro* by inhibiting class III PI3-kinase (18). In the current study, treatment with 3-MA weakened the effect of LG in enhancing autophagy and reducing lipid accumulation. RAPA, as an inhibitor of mTOR, activates autophagy (18). Our study showed that treatment with LG enhanced autophagy and reduced lipid accumulation in steatotic L-02 cells, which was similar to RAPA. Therefore, the activation of autophagy plays a significant role in the anti-steatosis effect of LG *in vitro*.

Two limitations of the present study need to be addressed. Firstly, as the nature of NAFLD has been unclear until now, it is difficult to exactly differentiate simple hepatic steatosis from NASH. L-02 cell steatosis induced by exposure to FFA mixture may not always mimic simple hepatic steatosis. Secondly, as a result of the complexity in measuring autophagy, certain experiments are warranted, such as modulating the intensity of autophagy by knockdown or knockout of ATG genes.

In conclusion, the cell model, built by incubating L-02 cells with an FFA mixture (OA:PA ratio, 2:1) for 24 h, replicates the pathological features of human simple hepatic steatosis. Treatment with LG reduces lipid accumulation in steatotic L-02 cells and the activation of autophagy plays a significant role in the process. Thus, our data indicate that GLP-1 analogs are a promising treatment approach and the activation of autophagy may be a potential mechanism to improve hepatic steatosis.
